# Three-year trends and seasonal variation in patient satisfaction with primary health care services in Saudi Arabia: results from the national patient experience measurement program (2022–2024)

**DOI:** 10.3389/fpubh.2025.1727345

**Published:** 2026-01-14

**Authors:** Motab Aljohani

**Affiliations:** Department of Public Health, College of Health Science, Saudi Electronic University, Riyadh, Saudi Arabia

**Keywords:** health services quality, patient satisfaction, primary health care, Saudi Arabia, seasonal variation

## Abstract

**Background:**

Patient satisfaction is a key metric for assessing quality and equity in health systems. In Saudi Arabia, the Ministry of Health (MOH) has prioritized monitoring patient experiences to guide primary health care (PHC) improvements. Understanding seasonal variation in satisfaction is particularly important, as it can reveal fluctuations in service performance and inform workload planning, staffing, and quality improvement policies. This study examined three-year trends and seasonal variation in patient satisfaction with PHC services from 2022 to 2024.

**Methods:**

A retrospective repeated cross-sectional analysis was conducted using secondary data from the MOH Patient Experience Measurement Program. Data included responses from patients or their caregivers who visited PHC centres and completed the Press Ganey®-based patient satisfaction survey. Quarterly datasets from January 2022 to December 2024 across all PHC centres managed by 20 health clusters in Saudi Arabia were merged. The main outcome was the mean satisfaction score (scaled 0–100). Linear regression models were used to examine changes in patient satisfaction, adjusting for sociodemographic factors such as age, sex, and nationality. Interaction terms were introduced separately to assess temporal trends across different subgroups. Predicted margins were calculated and visualized to interpret subgroup patterns.

**Results:**

A total of 2,173,518 responses were analysed. The overall mean satisfaction score was 82.2 (SD = 23.4), increasing significantly from 79.7 in 2022 to 84.3 in 2024, *p* < 0.001. Older adults and non-Saudis reported higher satisfaction, while younger respondents and females had lower scores. The highest adjusted gain occurred in the last quarter of 2024 (*β* = 4.18; 95% CI: 4.01, 4.34). All subgroups showed upward trends, although disparities by age, sex, and nationality persisted throughout the three-year period.

**Conclusion:**

Patient satisfaction with PHC services in Saudi Arabia improved significantly between 2022 and 2024, with evidence of seasonal patterns. These findings emphasize the importance of using national patient experience data to strengthen patient-centered reforms, promote equity, and guide evidence-based PHC quality improvement and policy design.

## Introduction

Primary healthcare (PHC) represents the foundation of healthcare systems and serves as the first and most frequent point of contact for patients (1). Ensuring a positive patient experience during this time is central to promoting effective, equitable, and responsive health services. In recognition of this, patient satisfaction has gained prominence as a key performance metric, reflecting both perceived healthcare quality and patient-centeredness of care delivery (2, 3). Internationally, analyses of patient satisfaction trends over time have revealed that structural reforms, funding changes, and service innovations can have both immediate and long-term impacts on public perceptions toward health services (4, 5). Yet, satisfaction may also respond to more subtle or gradual influences such as staffing levels, service continuity, and patient expectations (6–8).

In recent years, Saudi Arabia has made significant investments to transform PHC as part of the broader national reforms outlined in Vision 2030 (9). Reforms have prioritized digital health expansion, workforce development, and appointment systems, supported by the systematic collection of patient feedback to guide continuous service improvement. The Ministry of Health’s (MOH) Patient Experience Measurement Program was introduced toward the end of the year 2020, to support this goal and provides routine national-level data on patient satisfaction (10, 11). These data offer a unique opportunity to assess the performance of PHC services over time and across diverse population groups.

Evidence from studies conducted in PHC settings in Saudi Arabia indicates that satisfaction with healthcare providers’ interactions is associated with improved patient experiences and greater adherence to treatment plans (12). In this Gulf country, the key drivers of patient satisfaction include the availability and accessibility of services, effective communication, rational conduct, technical competence, and the personal attributes of healthcare providers (13). However, ongoing concerns related to service delays, facility conditions, and prolonged waiting times continue to negatively impact overall satisfaction in Saudi Arabia (14–16).

**Figure 1:**
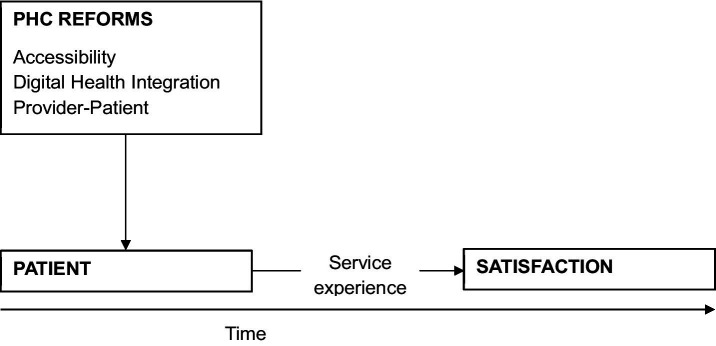
Conceptual model depicting how PHC reforms may shape patient expectations, service experiences, and satisfaction outcomes over time through changes in accessibility, digital health integration, and provider–patient interactions.

While previous studies in Saudi Arabia have provided point-in-time estimates of patient satisfaction, with overall scores ranging from 61.9 to 83.8% (12, 17), there is limited evidence on how satisfaction levels have changed nationally over time. This study builds on previous studies by employing quarterly data across a three-year period to capture temporal shifts in satisfaction and by disaggregating results across age, sex, and regional subgroups, areas rarely explored in prior research. Understanding these trends is essential to capture how patient perceptions respond to ongoing healthcare reforms, policy changes, and service delivery improvements (Figure 1). Additionally, little is known about seasonal variations in patient satisfaction or differences across demographic subgroups. Yet, seasonal variation may influence patient satisfaction due to fluctuations in service demand during national holidays and the Hajj season, when PHC centres often experience increased patient loads and staffing constraints. Examining these temporal dynamics can reveal system responsiveness during high-demand periods. This study addresses these existing gaps by providing new evidence from a rapidly evolving health system that may have broader international significance for enhancing primary health care (PHC) performance and patient-centeredness.

Therefore, three-year quarterly national patient satisfaction data from 2022 to 2024 were analysed to assess changes in satisfaction levels over time, examine seasonal variation, and explore subgroup differences in these trends by key population demographics in light of Saudi Arabia’s dynamic healthcare transformation agenda. Understanding changes in satisfaction patterns has significant implications for practice. Recognizing seasonal or subgroup differences in satisfaction can help health systems anticipate periods of lower patient experience, optimize workforce deployment, and allocate resources more effectively. In Saudi Arabia, these findings will inform the ongoing transformation of PHC. Internationally, they provide valuable lessons for countries seeking to enhance patient-centered primary care through the systematic use of patient feedback.

## Methods

### Study design and setting

This study used a retrospective repeated cross-sectional design based on secondary data obtained from the Ministry of Health (MOH) Patient Experience Measurement Program in Saudi Arabia. The data were drawn from the national Patient Experience Survey, which is conducted routinely across all government-run PHC centres in the country. The survey aims to collect patient feedback on the quality of care provided at PHCs as part of a broader initiative to enhance service delivery within the Saudi healthcare system.

The survey was based on the validated Press Ganey® model, culturally adapted for the Saudi context under Ministry of Health (MOH) oversight. The dataset included patient responses collected continuously from January 2022 to December 2024 across all 20 health clusters, which collectively manage 2,311 PHC centres serving diverse urban and rural populations (18). The MOH launched the Patient Experience Measurement Program in 2020 to standardize national evaluations of patient-centered care (11, 15). The Patient Experience Measurement Program includes multiple built-in quality control processes to ensure the accuracy and reliability of collected data. Survey responses are automatically validated at the point of submission through electronic checks for completeness, internal consistency, and duplicate entries. Quarterly data undergo routine audits and verification by the MOH before release. These standardized quality assurance procedures ensured that data analysed in this study met national reporting standards and maintain comparability across clusters and time periods. A three-year period (2022–2024) was selected to capture post-COVID-19 service trends and ensure stable quarterly comparisons through a repeated cross-sectional design.

### Participants and data collection

The study population consisted of adult patients aged 18 years and older who visited a PHC and subsequently responded to the MOH Patient Experience Survey. Patients were selected through a systematic process in which a random subset of visitors was invited to participate via text messages sent to their mobile phones within 24 h of their clinic visit. Invitations were sent daily, and participants were given up to 14 days to complete the survey online. The survey was voluntary, and completion was taken as implied consent. The response rate for this survey is reportedly satisfactory at 76.5% (15), which may enhance the representativeness and reliability of the findings. For this analysis, only fully completed surveys with valid satisfaction score data were included, while any observation with missing outcome data was excluded. Because fewer than 0.01% of observations lacked valid satisfaction scores, no imputation was performed; those cases were listwise excluded.

### Variables

The survey instrument included 22 questions assessing patients’ experiences during their most recent PHC visit. These questions were grouped into seven domains: registration, appointment scheduling, movement through the clinic, experiences with nurses, interactions with care providers, personal and environmental issues, and overall patient perceptions. Each question was rated on a 5-point Likert scale ranging from 1 (not at all satisfied) to 5 (extremely satisfied). The 0–100 satisfaction scores were generated through the proprietary Press Ganey® algorithm used by the MOH across all survey years, ensuring methodological consistency and comparability of scores over time. The algorithm included converting the 5-point Likert scale scores to a 0–100 scale using the formula: (score − 1) × 25. The main outcome variable was the mean satisfaction score, calculated as an overall average across all domains. Domain means were first averaged at the individual level before being aggregated across quarters and demographic subgroups for analysis.

In addition to the satisfaction outcome, patient-level covariates included in the dataset were age category (≤40, 41–60, and >60 years), gender (male or female) and nationality group (Saudi or non-Saudi), as self-reported by the respondents. Other variables generated during analysis included: survey period (defined quarterly from Q1_2022 to Q4_2024) and survey year (2022, 2023, 2024).

### Data analysis

Data were analysed using Stata version 16.1 (StataCorp, College Station, TX, USA). All quarterly datasets from 2022 to 2024 were appended and cleaned to ensure consistency in variable formats and coding. A new variable was generated to capture the survey period, combining year and quarter (e.g., Q1_2022 to Q4_2024), and used to assess temporal trends. Quarterly datasets were merged without additional weighting, as each cluster’s response volume was proportional to its service catchment size based on the MOH sampling framework.

Descriptive statistics, including means and standard deviations, were calculated for satisfaction scores across survey years and subgroups. To account for changes over time, a multiple linear regression model was fitted with mean satisfaction score as the dependent variable and survey period as the primary predictor. Covariates included age group, sex and nationality. Satisfaction scores were treated as continuous variables ranging from 0 to 100, consistent with Press Ganey® scoring methods. A multiple linear regression model was applied because the data represent independent quarterly cross-sections rather than repeated measures of the same respondents. Mixed-effects or time-series models that assume autocorrelation or within-cluster dependency were therefore not appropriate. Linear regression model assumptions were also evaluated through a series of diagnostic checks. Normality of residuals was assessed visually using Q–Q plots and Shapiro–Wilk test, while homoscedasticity was examined using residuals-versus-fitted plots. Multicollinearity was assessed using the variance inflation factor (VIF), and the mean VIF across all covariates was 2.0, confirming that multicollinearity was not a concern in the analysis. No additional sensitivity analyses were conducted, as these diagnostic results and the large, nationally representative sample supported the robustness and stability of the model estimates.

Interaction terms between survey period and each subgroup variable (e.g., age group, sex) were introduced in separate models to explore differential trends. Post-estimation margins and marginsplot commands were used to generate adjusted mean satisfaction scores and visualize trends over time with 95% confidence intervals. Plots were customized to reflect differences across subgroups, with error bars and value labels for interpretability.

Given the consistently large and balanced sample sizes across survey quarters, and the standardized national survey methodology, no additional sampling weights were applied. To address potential differences in respondent characteristics over time, all models were adjusted for key demographic covariates, including age, sex, and nationality.

## Results

### Characteristics of study participants

A total of 2,173,518 participant responses were included in the study after removing 169 respondents with incomplete data on the main outcome (response rate = 99.9%). The overall mean satisfaction score across the three-year period was 82.2 (SD = 23.4), with a significant annual increase observed from 79.7 (SD = 24.9) in 2022 to 81.7 (SD = 23.7) in 2023, and reaching 84.3 (SD = 21.9) in 2024, *p* < 0.001. As shown in Table 1, slightly more than half of the respondents were female (50.6%), and the majority were Saudi nationals (92.3%). Most respondents were younger than 40 years (67.2%), while 26.6% were aged between 41 and 60 years and only 6.2% were above 60 years. The number of survey respondents increased gradually across the quarters from Q1-2022 to Q4-2024.

**Table 1:** Characteristics of the survey participants.

Sub-group	Frequency (n)	Percentage (%) or (mean ± SD)
Overall satisfaction scores	2,173,518	82.2 ± 23.41
Sex		
Male	1,073,091	49.37
Female	1,100,427	50.63
Nationality		
Saudi Arabia	2,005,863	92.29
Others	167,655	7.71
Age category (years)		
Less than 40	1,461,103	67.22
Between 41 and 60	578,770	26.63
Above 60	133,645	6.15
Survey period (Quarter and Year)		
Q1-2022	118,706	5.46
Q2-2022	124,133	5.71
Q3-2022	158,343	7.29
Q4-2022	155,647	7.16
Q1-2023	174,630	8.03
Q2-2023	162,626	7.48
Q3-2023	205,085	9.44
Q4-2023	212,915	9.80
Q1-2024	235,877	10.85
Q2-2024	202,393	9.31
Q3-2024	211,950	9.75
Q4-2025	211,213	9.72

### Overall trends in patient satisfaction over time

**Figure 2:**
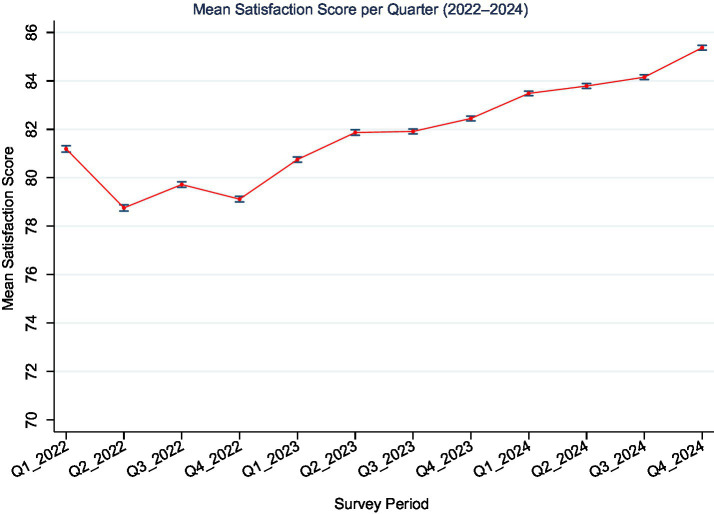
Adjusted mean satisfaction scores (0–100 scale) with 95% confidence intervals across 12 quarterly periods from Q1-2022 to Q4-2024. A general upward trend in satisfaction was observed over time.

As shown in Figure 2, the trend in patient satisfaction revealed modest seasonal fluctuations and a general upward trajectory in mean satisfaction scores across the survey periods. The lowest satisfaction scores were observed during the early quarters of 2022, while notable improvements were observed in subsequent years, particularly in 2024.

### Predictors of satisfaction with primary healthcare services

Findings from the multiple linear regression analysis (Table 2) indicated that patient age, sex, nationality, and survey period were significantly associated with satisfaction scores (all *p*-values < 0.001). The *β* coefficients represent point differences in the mean satisfaction score (0–100 scale) compared to the reference category after adjusting for all other covariates. Compared to respondents aged <40 years, those aged 41–60 and >60 years reported significantly higher satisfaction scores by 5.35 (95% CI: 5.28 to 5.42) and 6.82 (95% CI: 6.69 to 6.95) points, respectively. Female respondents reported slightly lower satisfaction scores than males (*β* = −2.07, 95% CI: −2.13 to −2.01), and Saudi nationals also had lower mean satisfaction scores compared to non-Saudis (*β* = −4.21, 95% CI: −4.33 to −4.09).

**Table 2:** Multiple linear regression showing differences in mean satisfaction scores across different predictor variables.

Predictor variable	Coefficient	95% C. I	*p*-value
Age category (years)			
Less than 40	Ref		
41–60	5.346	5.276, 5.417	<0.001
>60 years	6.820	6.689, 6.950	<0.001
Sex			
Male	Ref		
Female	−2.068	−2.130, −2.006	<0.001
Nationality			
Other			
Saudi Arabia	−4.209	−4.325, −4.094	<0.001
Survey period			
Q1_2022	Ref		
Q2_2022	−2.444	−2.628, −2.260	<0.001
Q3_2022	−1.473	−1.647, −1.299	<0.001
Q4_2022	−2.075	−2.250, −1.901	<0.001
Q1_2023	−0.446	−0.616, −0.276	<0.001
Q2_2023	0.677	0.504, 0.850	<0.001
Q3_2023	0.721	0.556, 0.886	<0.001
Q4_2023	1.254	1.090, 1.419	<0.001
Q1_2024	2.292	2.131, 2.453	<0.001
Q2_2024	2.595	2.429, 2.761	<0.001
Q3_2024	2.958	2.793, 3.122	<0.001
Q4_2024	4.176	4.012, 4.341	<0.001
Model R^2^	0.259		

Temporal comparisons with Q1-2022 as the reference showed significant quarter-to-quarter differences. While satisfaction was slightly lower during Q2–Q4 of 2022, scores began to rise from Q2-2023 onward, with the highest adjusted increase observed in Q4-2024 (*β* = 4.18, 95% CI: 4.01 to 4.34).

Box 1 Policy implications and recommendations.

**Table:** 

Key Finding 1: A 4-point national improvement in satisfaction (2022–2024) Implication: PHC reforms are perhaps yielding measurable gains. Recommendation: Sustain digital transformation and staff training initiatives. Key Finding 2: Persistent subgroup disparities (age, sex, nationality) Implication: Targeted strategies are needed to ensure equitable care. Recommendation: Develop gender-sensitive and culturally adaptive PHC models. Key Finding 3: Seasonal variation in satisfaction Implication: Service quality fluctuates with workload and staffing cycles. Recommendation: Align workforce deployment and communication strategies with peak periods. Key Finding 4: Large national survey infrastructure/data Implication: Enables real-time performance monitoring. Recommendation: Institutionalize patient experience dashboards within MOH quality systems.

### Adjusted trends in satisfaction over time

**Figure 3:**
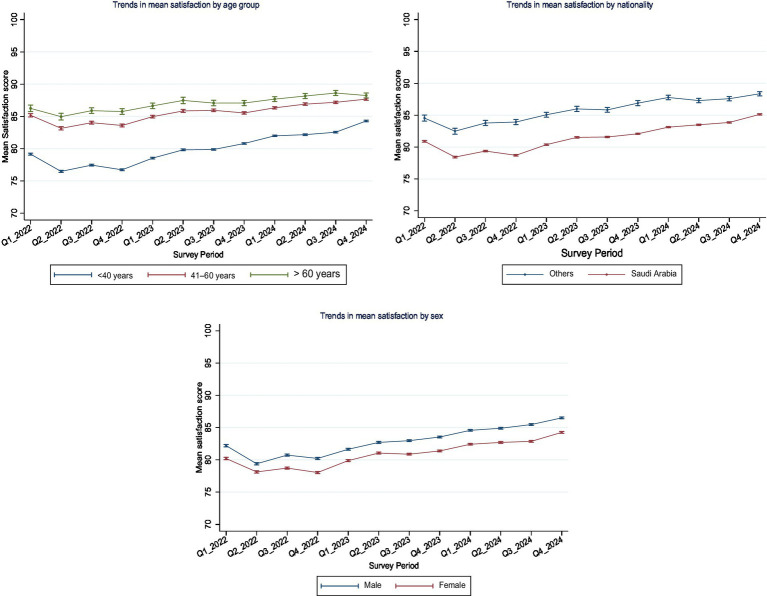
Adjusted mean satisfaction scores (0–100 scale) with 95% confidence intervals by age, sex, and nationality subgroups from Q1-2022 to Q4-2024. Satisfaction increased across all subgroups, though disparities persisted.

Across all subgroups, adjusted satisfaction scores demonstrated a general upward trend over the 12 survey periods from Q1-2022 to Q4-2024 (Figure 3). Interaction effects between survey year and demographic variables were statistically significant (*p* < 0.05), indicating that satisfaction trajectories varied across subgroups as follows.

Age group: satisfaction was highest among respondents aged >60 years, followed by those aged 41–60, and lowest among those <40 years. All age groups showed gradual increases in satisfaction over time, with older age consistently associated with higher scores.

Sex: males reported higher satisfaction scores than females across all survey periods. However, both groups experienced progressive increases, with the gap between them remaining relatively stable.

Nationality: non-Saudi participants reported markedly higher satisfaction scores than Saudi nationals throughout the study. Although both groups showed improvements, the disparity persisted across all quarters.

## Discussion

This study aimed to assess the trends and seasonal variation in patient satisfaction with PHC services in Saudi Arabia over a three-year period from 2022 to 2024, using data from the Ministry of Health’s Patient Experience Measurement Program. The analysis revealed a steady significant improvement in overall satisfaction scores. Notably, the highest increase was observed in the final quarter of 2024. Although satisfaction improved across all demographic groups, persistent disparities by age, sex, and nationality were evident.

The overall 4-point increase in satisfaction scores between 2022 and 2024 may appear modest in absolute terms but it represents a meaningful improvement at the population level, particularly given the large national sample. In Press Ganey® benchmarking, a 3–5 point gain over consecutive years is typically interpreted as a substantial improvement in patient experience, supporting the policy relevance of these findings for PHC performance monitoring.

This study has several strengths. It leverages one of the largest and most comprehensive patient satisfaction datasets available in the country, encompassing over two million responses collected from all 20 health clusters across all regions in Saudi Arabia. The use of a standardized, validated survey tool from Press Ganey® and adapted to the local context enhances the reliability and comparability of the findings across similar studies.

However, some limitations should be acknowledged. First, the use of self-reported satisfaction data introduces potential response bias. Increases in satisfaction may partly reflect evolving public expectations or improved familiarity with digital survey systems rather than purely service-level improvements. Future mixed-method studies combining patient feedback with objective quality indicators are recommended to disentangle perceptual from performance-driven change. In the same line, the mode of survey administration, i.e., through SMS invitations to complete an online questionnaire, may introduce response and selection biases. Individuals with limited digital access, lower literacy, or older age may have been underrepresented, potentially influencing subgroup differences observed in satisfaction scores. Although the overall response rate for this survey is reportedly high (76.5%) (15), this approach may still favor respondents who are more technologically engaged or health literate. Consequently, satisfaction levels could be slightly overestimated compared to the broader PHC user population. These factors should be considered when interpreting the findings, as they may confound observed associations between demographic subgroups and satisfaction outcomes. Future research employing mixed survey modes or targeted outreach to digitally excluded populations would enhance representativeness and reduce potential bias. Second, although the data are collected continuously, the design remains cross-sectional in nature, meaning that changes in satisfaction cannot be causally attributed to specific reforms or interventions. Third, the dataset lacks information on clinical conditions, service utilization patterns, or facility-level characteristics, which may influence satisfaction and limit the ability to control for case mix or provider variation. Although key linear regression assumptions were checked and met, formal sensitivity analyses were not conducted. Future research may incorporate alternative modeling approaches or robustness checks to further validate these findings. Finally, while the analysis adjusts for age, sex, and nationality, other relevant factors such as socioeconomic status, health literacy, or region of residence were not captured by the survey and may influence satisfaction patterns.

The improvement in patient satisfaction observed during the study period coincides with a series of PHC reforms launched under Saudi Arabia’s Vision 2030 health transformation program. These reforms have included investments in digital health platforms, efforts to improve appointment scheduling and access, expansion of virtual consultations, and restructuring of service delivery models through regional health clusters (19–21) which may have contributed to the broader context in which satisfaction improved. The period from 2022 to 2024 also saw the continuation of the Patient Experience Measurement Program as a key tool for performance monitoring and accountability. These concurrent changes may help explain the progressive rise in satisfaction scores over time. However, as factors such as health literacy, socioeconomic status, and service accessibility may also influence patient satisfaction, interpretations linking PHC reforms to improvements in satisfaction should be made cautiously, especially considering the cross-sectional design of this study, which precludes causal inference. Similarly, studies from the UK, for example, have shown that satisfaction may rise or fall in response to reforms such as funding shifts or government healthcare expenditure (2, 22). The steady improvement in satisfaction observed in this study aligns with such literature and suggests that reforms in PHC service delivery in Saudi Arabia may be yielding measurable gains in patient experience. However, the persistence of subgroup disparities particularly among younger patients, females, and Saudi nationals indicates that the benefits of reform may not be evenly distributed. Targeted efforts are needed to address these gaps through culturally sensitive communication, gender-responsive care models, and services that resonate with the expectations of diverse population groups (23).

The seasonal fluctuations in satisfaction observed across quarters also point to the importance of operational readiness and adaptive service planning (24, 25). Holiday periods, Hajj seasons, or other high-demand intervals may influence staffing and performance, which in turn affects patient perception (26). Specifically, satisfaction tended to dip during Q2 and Q4, which coincides with peak summer and Hajj seasons when staff rotations, increased workloads, and temporary service reorganization may affect patient flow and waiting times (27, 28). Conversely, post-Hajj quarters often showed recovery in satisfaction, potentially due to normalization of workloads and administrative adjustments. These fluctuations demonstrate the operational importance of aligning staffing, logistics, and patient communication strategies with known seasonal demand cycles. Also, embedding real-time patient feedback into routine planning cycles can help PHC centres respond more effectively to changing service pressures and sustain quality improvements throughout the year. For instance, in the United Kingdom, Carter et al. highlighted the importance of aligning feedback mechanisms with existing patient and practice routines to improve engagement and support adaptive service planning (29). These findings reinforce the need to integrate timely patient feedback into routine planning processes to enhance the responsiveness and quality of PHC services throughout the year.

The persistence of satisfaction differences by age, sex, and nationality over the three-years warrants closer attention. Older adults reported consistently higher satisfaction scores than younger respondents, a pattern that may reflect differences in expectations, health needs, or frequency of service use (30–32). Older patients may also have longer-standing relationships with care providers or may place greater value on continuity and attentiveness, factors that positively influence perceptions of care (33, 34). In contrast, younger patients may have higher expectations for convenience, responsiveness, or digital engagement, and may be more critical when these expectations are unmet (35). A similar pattern has been observed in the UK, where older adults reported higher satisfaction levels than their young counterparts over time (2).

The observed gender gap, with females reporting lower satisfaction than males, echoes findings from other studies in both regional (14, 17) and international contexts (36). Possible explanations include differences in communication preferences, staff preferences, perceived attentiveness from providers, or unmet needs related to reproductive or family health services (37). Additionally, societal or cultural factors may influence how women experience and report care, particularly in settings where gender norms shape interactions with the health system such as in Saudi Arabia (38).

Non-Saudi patients consistently reported higher satisfaction than Saudi nationals. It can be speculated that this difference may stem from variations in expectations, language comfort, or perceived gratitude for access to subsidized services. Alternatively, Saudi patients, as the predominant population group, may evaluate public healthcare services against higher expectations or in comparison with private sector alternatives. Supporting this concept, a study by Alodhialah et al. reported that private healthcare facilities outperformed public ones in terms of patient satisfaction and loyalty (39), which could indicate that Saudi patients are more discerning in their evaluations due to greater exposure to varied service options or higher baseline expectations. Regardless of the underlying causes, the persistence of these subgroup disparities exhibits the need for a more tailored approach to service evaluation and design that accounts for the experiences and preferences of diverse patient populations.

These findings have important implications for health policy and PHC service planning in Saudi Arabia (Box 1). The observed improvement in satisfaction may suggest that recent investments and reforms in PHC such as digital transformation, service integration, and enhanced patient engagement are resonating positively with service users. Routine collection and analysis of patient experience data, as demonstrated by the MOH Patient Experience Measurement Program, offer a valuable mechanism for monitoring system performance and guiding quality improvement efforts.

While this study reflects patient satisfaction trends within the Saudi PHC context, its implications may extend to other Gulf and possibly low- and middle-income country settings implementing similar health reforms. The use of standardized patient experience tools, digital feedback systems, and decentralized health clusters demonstrate similar global trends in PHC transformation. Thus, the patterns observed in this study, as well as the progressive improvement in patient satisfaction with persistent subgroup disparities, may inform the design and monitoring of patient-cantered reforms in comparable health systems. Future iterations of the Patient Experience Survey could benefit from including additional demographic and clinical variables to enable more comprehensive analyses of factors influencing patient satisfaction.

## Conclusion

The study provides national evidence of a steady improvement in patient satisfaction with PHC services in Saudi Arabia between 2022 and 2024. While these gains are encouraging and may reflect the positive impact of ongoing PHC reforms, persistent disparities across age, sex, and nationality groups highlight the need for more targeted efforts to promote equity in patient experience. Seasonal variation in satisfaction also suggests opportunities for system-level responsiveness to changing service demands. Continued investment in patient-centred care, coupled with the routine use of national satisfaction data, will be essential to sustaining improvements and guiding future policy development within the evolving Saudi healthcare system. Future research should explore how changes in patient satisfaction relate to health outcomes, service utilization, and facility-level performance indicators. Linking satisfaction data with clinical or administrative datasets could reveal whether improved patient experiences translate into measurable gains in care quality and efficiency. Qualitative studies are also warranted to understand the underlying drivers of satisfaction differences across demographic and cultural groups.

## Data Availability

The data analyzed in this study is subject to the following licenses/restrictions: the dataset used in this study was obtained from the Ministry of Health (MOH) Patient Experience Measurement Program in Saudi Arabia. These data are not publicly available due to privacy and confidentiality agreements. Access to the dataset is restricted to authorized researchers through the MOH upon formal request and approval. Only aggregated and anonymized results are presented in this manuscript. Requests to access the dataset can be made through the Ministry of Health (MOH) Patient Experience Measurement Program, Saudi Arabia. Website: https://www.moh.gov.sa Email: Data-Office@moh.gov.sa.
